# Kisspeptin is elevated in the brain after intracerebral haemorrhagic stroke

**DOI:** 10.1038/s41598-024-83514-0

**Published:** 2024-12-30

**Authors:** Saumya Maheshwari, In Hwa Um, Struan Donachie, Nafeesa Asghar, Karina McDade, Tracey Millar, David J. Harrison, Javier A. Tello

**Affiliations:** 1 School of Medicine, University of St Andrews, Medical and Biological Sciences Building, North Haugh, St Andrews, UK; 2 College of Medicine and Veterinary Medicine, University of Edinburgh, Edinburgh, UK; 3 Academic Neuropathology, The University of Edinburgh, Edinburgh, UK; 4 Biomedical Research Complex, University of St Andrews, St Andrews, UK; 5 Centre for Biophotonics, University of St Andrews, North Haugh, St Andrews, UK

**Keywords:** Stroke, Intracerebral haemorrhage, ICH, Kisspeptin, Biomarker, Cerebral amyloid angiopathy, CAA, Biomarkers, Translational research, Neuro-vascular interactions

## Abstract

Intracerebral haemorrhage (ICH) is the most severe subtype of stroke, with a 2-year mortality of nearly 50% and the greatest rate of disability amongst stroke survivors. Whilst treatment options for ICH remain limited, the condition requires prompt identification and rapid intervention to reduce permanent brain damage, with diagnosis traditionally confirmed by CT imaging. Although imaging is excellent at determining the presence of an intracranial bleed, biomarkers may help to identify the type of stroke or when the stroke began. Kisspeptin is a neuropeptide best known for its functions in reproductive biology, but recent preclinical studies have demonstrated that kisspeptins are upregulated in rodent models of haemorrhagic stroke. Here we report for the first time that kisspeptin immunoreactivity is significantly higher in post-mortem human brain tissue after both ICH and ICH associated with cerebral amyloid angiopathy. Machine learning and artificial intelligence-enabled image analysis of multiplexed immunolabeled brain tissues demonstrated that kisspeptin immunoreactivity was higher in cells of the microvasculature (CD105+), but not in neurons or astrocytes when compared to controls. Further spatial analysis indicated that kisspeptin immunoreactivity was concentrated to the region of haemorrhage. These results indicate that following ICH, kisspeptin is significantly higher in the human brain, suggesting expression from local vasculature or recruitment to the haematoma. Further work is required to determine the biological mechanisms underlying kisspeptin elevation within the ICH microenvironment and its potential utility as a novel biomarker or therapeutic target for ICH.

**Supplementary Information:**

The online version contains supplementary material available at 10.1038/s41598-024-83514-0.

## Introduction

Intracerebral haemorrhage (ICH) is a subtype of haemorrhagic stroke characterised by the rupture of blood vessels and bleeding into the parenchyma of the brain. The incidence of haemorrhagic stroke is second only to ischemic stroke, accounting for 10–20% of all new strokes every year^1^. Between 80–85% of haemorrhagic strokes are considered primary ICH caused by the spontaneous rupture and bleeding of blood vessels usually weakened by amyloid angiopathy or chronic hypertensive damage. Secondary ICH (15–20%) has a multitude of causes including stimulant drugs, tumours, vascular malformation and coagulopathies^2^. ICH strokes are less common than ischemic strokes but they are far more severe, with a one-month mortality rate of 35–40% increasing to 54% after one year^3,4^.

ICH results in severe brain damage due to a combination of primary and secondary injury. The primary injury arises from the immediate physical disruption caused by increased hydrostatic pressure due to blood emerging from ruptured vessels. This leads to elevated intracranial pressure, resulting in insult to neurons and tissue damage. Secondary injury occurs due to oedema and the build-up of clot-derived neurotoxic factors inducing neuroinflammation and eventual cell death, days to months after the initial bleed. Key toxic factors responsible for driving the process of secondary brain injury include blood components that contribute to oxidative and inflammatory pathways including haemoglobin, hemin, and free iron^5^.

Considerable progress has been made in understanding the pathological mechanisms underlying ICH, which has led to the incidence of ICH beginning to fall largely due to controlling risk factors, especially arterial hypertension, and improved diagnosis of secondary ICH^6^. However, the fatality rates (within 48 h of stroke) have not improved in more than 25 years pointing to the need for more rapid diagnosis and treatments^7^. Although most strokes occur in the elderly, anyone is at risk of stroke at any time. In fact, approximately a quarter of all strokes in the UK occur in people of working age.

The presence of cerebral amyloid angiopathy (CAA) increases the risk of both lobar and recurrent ICH. CAA is characterised by the progressive deposition of beta-amyloid in the linings of small-to-medium sized vessels in the cerebral cortex and overlying leptomeninges^8,9^. The deposition of amyloid fibrils results in fragile vessels primarily associated with cerebral microbleeds, but recent studies have demonstrated that CAA triggers extensive ischemic alterations, including cortical microinfarcts and white matter hyperintensities, which are linked to cognitive decline^10^. CAA is predominantly seen as a sporadic disease in the elderly, increasing in incidence and severity with age and there is some overlap with Alzheimer’s disease pathology^8^. A few genetic mutations result in a pure form of CAA, found in younger patients typically associated with more severe clinical symptoms and cognitive dysfunction^11^.

Stroke diagnosis and treatment is a challenging and time-sensitive issue. To further complicate matters, there are several conditions known as stroke mimics which often confuse acute stroke diagnosis. Stroke mimics are a group of disorders that present similarly to stroke clinically, but without a characteristic tissue infarction. Stroke mimics account for up to 40% of all acute stroke unit admissions^12^. Examples of conditions that can mimic stroke include seizures, sepsis, tumours and psychiatric disorders. Although bedside clinical assessments, such as neurological signs and symptoms, aid physicians in differentiating stroke types, the current gold standard for diagnosing and distinguishing between stroke types is a non-contrast computed tomography (CT) scan^13^. However, the use of CT scans requires exposure to ionising radiation and expert radiological evaluation, which consumes valuable time within the critical treatment window. The development of a new clinical tool capable of aiding physicians in diagnosing stroke independently of CT scans could expedite stroke treatments and improve patient outcomes.

A biomarker test that enables a rapid diagnosis and identification of stroke subtype would be extremely beneficial in aiding stroke management. Recently, several studies have investigated the upregulation of various inflammatory markers or proteins following stroke in the hope of developing biomarker panels which provide an accurate prognostic or diagnostic test for stroke. Although some of these proteins have shown promise, most studies have failed to identify a molecular marker which enables an accurate diagnosis of stroke in an acute setting. As our understanding of stroke pathology grows, it is hoped that a novel protein involved in the stroke response may be identified and used to develop an objective test which is specific and sensitive enough to diagnose and distinguish between different stroke subtypes. Such a test could fast-track patient treatment into focused stroke care pathways and may save limited resources by identifying those who do not require emergency transfers or admission to specialised stroke units.

Kisspeptins are a family of peptide hormones encoded by the *KISS1* gene, and are ligands for a G protein coupled receptor, KISS1R, formerly known as GPR54. *KISS1* is translated into a 145 amino acid precursor protein which is enzymatically processed into a range of peptides that all share a common C-terminal decapeptide core that is required for receptor binding. The largest family member is a 54-amino acid peptide, and further processing produces 14-, 13-, or 10-amino acid peptides. Kisspeptins are highly expressed in the central nervous system and are recognised for their critical role in promoting puberty and regulating reproduction^14–17^. The presence of kisspeptins and KISS1R in the human vasculature was first reported in 2007 by Mead and colleagues, who established that kisspeptins had vasoconstrictive properties ex vivo^18^. In a recent preclinical rat study, kisspeptins and Kiss1r were found to be elevated after subarachnoid haemorrhage (SAH)^19^.

To the best of our knowledge, no studies have yet linked kisspeptin with stroke pathology in humans. Thus, determining if ICH in humans is associated with increased kisspeptin levels may identify a novel biomarker to help facilitate rapid stroke monitoring. In the current study, to investigate whether kisspeptin was higher in the brain following haemorrhagic stroke, we tested post-mortem brain tissue from individuals who suffered ICH, ICH associated with CAA, and control subjects who died suddenly from other causes. We used multiplexed immunolabelling, supported by machine learning and artificial intelligence image analysis, to identify whether kisspeptin localised to neurons, astrocytes or endothelial cells of the microvasculature. We also conducted spatial image analysis to understand the distribution of kisspeptin (KISS) immunoreactivity (-ir) in regions of haemorrhage.

## Methods

### Ethical approval and patients

Brain tissue was obtained from The Edinburgh Brain Bank^20^, under the NHS Research Ethics Committee approval (REC reference 21/ES/0087). The Committee is constituted in accordance with the Governance Arrangements for Research Ethics Committees and complies fully with the Standard Operating Procedures for Research Ethics Committees in the UK. Informed consent was obtained from participants, or their legal representatives as required by the Human Tissue (Scotland) Act 2006. The study was granted local ethical approval from the University of St Andrews, School of Medicine Research Ethics Committee acting on behalf of the University Teaching and Research Ethics Committee (Code MD14617). In total, tissue from 17 donors was included in the current study (see Table 1 for individual subject characteristics/demographics). Samples for this study were collected post-mortem from patients who suffered either primary ICH or ICH due to CAA. Controls were post-mortem samples collected from patients who died suddenly from other causes. Tissue was coded and lacked identifiable patient details. Tissue was formalin-fixed, embedded into paraffin blocks, sectioned at 4 µm thickness and mounted onto Superfrost Plus slides. The presence of haemorrhage with or without CAA was histologically confirmed.

**Table 1:** Post-mortem sample subject characteristics.

Group	Days after ICH until death	Age	Sex	Area
ICH	2	46	M	Basal ganglia
ICH	3	63	F	Basal ganglia
ICH	6	64	F	Basal ganglia
ICH	2	71	M	Frontal BA46
ICH	10	77	M	Basal ganglia
ICH	4	82	M	Frontal BA46
ICH CAA	3	67	M	Periventricular WM
ICH CAA	18	72	M	Frontal BA46
ICH CAA	22	77	M	Frontal BA46
ICH CAA	1216	79	F	BA41/42
ICH CAA	3	87	M	Frontal BA46
Control	No ICH	46	M	Basal ganglia
Control	No ICH	63	M	Basal ganglia
Control	No ICH	71	F	Frontal BA46
Control	No ICH	71	M	Frontal BA46
Control	No ICH	82	F	Frontal BA46
Control	No ICH	84	M	Basal ganglia

Abbreviations: ICH—intracerebral haemorrhage; ICH CAA—intracerebral haemorrhage with cerebral amyloid angiopathy; M—male; F—female

### Study size

Given the absence of published studies investigating kisspeptin after ICH in humans, we did not have prior size estimates on which to base sample size calculations. Therefore, we included controls available to us from the Edinburgh Brain Tissue Bank with age and sex distributions similar to those of patients incident with ICH in our population and used a case-control ratio of approximately 2:1 ICH (patients who suffered primary ICH or ICH with CAA to sudden-death controls) to maximise power.

### Detecting Kisspeptin-immunoreactive elements using multiplex immunofluorescence (mIF) and image acquisition

KISS-ir in human tissue was assessed using a multiplex immunofluorescence labelling approach with a polyclonal anti-kisspeptin-10 (Kp-10) antiserum (Cat# AC564; a generous gift from Dr Alain Caraty, INRA, France) as described previously^21^. This antiserum was raised in rabbits against mouse Kp-10, which shares 90% sequence identity with humans. Controls in the present study included omission of the kisspeptin primary antiserum from the primary antibody incubation step and incubation with an equal concentration of rabbit IgG, either of which did not produce any immunolabelling. To ensure the specificity of the antiserum for human KISS-10, pre-adsorption controls using 100 µg/ml of human KISS-10 or a non-specific peptide (GnRH1) incubated with the anti-kisspeptin-10 antiserum (1:15,000 diluted in blocking buffer) overnight. Pre-adsorption with human KISS-10 significantly reduced anti-KISS immunolabelling whereas pre-adsorption with the non-specific peptide did not (see Supplementary Fig. 1).

To localise kisspeptin immunoreactivity to endothelial cells of the microvasculature, antibodies against both vascular endothelial markers CD105 (endoglin) and CD31 (PECAM-1) were used. Endoglin is expressed in the endothelium of all types of brain vessels^22^ but exhibits elevated expression in angiogenic cells and during active vascular remodelling^23^, whereas PECAM-1 is regarded as a non-inducible constitutive marker for vascular endothelial cells^24^.

To summarise the multiplex immunofluorescence procedure, paraffin-embedded tissue sections (4 µm thickness) mounted onto Superfrost Plus slides were heated to 60 °C for 1 h. Tissue sections were deparaffinised by immersion in xylene and rehydrated using a decreasing ethanol gradient (100%, 95%, 70% in PBS). Slides were then rinsed in PBS and immersed in PBS with 0.3% Triton X-100 for 10 min to permeabilise the tissue sections. This was followed by heat-induced epitope retrieval (HIER) by immersion in 10 mM sodium citrate pH 6.0 heated and maintained above 90 °C for 10 min using a microwave. Any remaining endogenous tissue peroxidase was quenched by immersion in 3% hydrogen peroxide diluted in methanol for 25 min. Slides were then rinsed in PBS. Following this, Blocking Buffer (BB; PBS containing 0.025% Triton X-100, 0.5% TSA blocking reagent [Cat no. FP1012, Perkin Elmer UK], 5% donkey serum) was applied to each section for 45 min. First, a mouse monoclonal anti-GFAP antibody (1:4000; Sigma Aldrich UK, G3893) diluted in BB, was applied to each section and incubated at 4 °C overnight. Sections were then washed three times in PBS with 0.05% Tween-20 (PBST) and incubated with secondary antibody, donkey anti-mouse IgG-peroxidase (1:650; Jackson ImmunoResearch Ltd., 715-036-150) diluted in BB at 37 °C for 45 min. Sections were then washed three times in PBST and GFAP-immunoreactivity was localised using TSA Plus Cyanine 5 (1:400; Akoya Biosciences US, #NEL745001KT) at 37 °C for 1 h. The slides were then washed three times in PBST and HIER was repeated for 10 min to remove tissue-bound primary and secondary antibodies. Any remaining peroxidase activity was quenched by immersion in 3% hydrogen peroxide diluted in PBS for 25 min. The slides were then rinsed in PBS and BB was applied to each section for 45 min. Next, rabbit anti-kisspeptin antibody (1:15,000; AC564) and chicken anti-MAP2 antibody (1:6,000; Invitrogen UK, PA1-10005) were diluted in BB and applied to each section at 4 °C overnight. Slides were then washed three times in PBST. To detect kisspeptin immunoreactivity, the sections were first incubated with donkey anti-rabbit IgG-peroxidase (1:650; Jackson ImmunoResearch Ltd., 711-036-152) at 37 °C for 45 min and then washed three times in PBST. Kisspeptin immunoreactivity was localised using TSA Plus Cyanine 3 (1:400; Akoya Biosciences US, #NEL744001KT) at 37 °C for 1 h. After incubation, the slides were washed to remove excess TSA Plus Cyanine 3. To inactivate the peroxidase complex added previously, the slides were quenched by immersion in 3% hydrogen peroxide diluted in PBS for 25 min. Slides were then washed in PBST twice. Next, the sections were incubated with donkey anti-chicken IgY-peroxidase (1:650; Jackson ImmunoResearch Ltd., 703-035-155) at 37 °C for 45 min. Sections were then washed three times in PBST and MAP2-immunoreactivity was localised using TSA Plus Fluorescein (1:400; Akoya Biosciences US, #NEL741001KT) at 37 °C for 1 h. After a wash in PBST, the slides were counterstained with Hoechst 33342 (1 µg/ml) for 10 min rinsed twice in PBST and mounted (Epredia Immu-Mount, Fisher Scientific, 9990412).

The Zeiss Axio Scan Z1 Digital Slide Scanner (Carl Zeiss Microscopy, Germany) was used to acquire whole slide fluorescence images at × 20 objective magnification and data was exported as .czi files. The same scanning profile was used to image the ICH, ICH CAA and sudden death control sections using a Plan-Apochromat 20x/0.8 M27 objective. This scanning profile was created using four different fluorescence channels, H3342 (348/455 nm), FITC (495/519 nm), Cy3 (548/561 nm), Cy5 (650/673).

### Immunohistochemistry for identification of CD105+ blood vessels

After acquiring images from the multiplexed immunofluorescence labelling, the slides were immersed in 0.1% TBST buffer at 40 °C overnight until the coverslips released. The slides were then transferred to the Leica Bond RX Autostainer and were treated with BOND epitope retrieval 1 (ER1) buffer (Leica, AR9961) at 95 °C for 20 min to remove all tissue bound primary and secondary antibodies. An immunohistochemistry protocol using Bond polymer refine detection kit (Leica, DS9800) was run on all sections, which included peroxide block for 5 min, primary antibody incubation (CD105, Human Protein Altas, HPA, HPA067440, 1:400 dilution) for 1 h, polymer secondary antibody for 30 min, mixed DAB chromogen for 10 min and haematoxylin (Leica, DS9800) for 5 min. The sections were then dehydrated using 50%, 80%, 100% alcohols, and xylene for clearing, followed by DPX (Cell Path, SEA-1304-00A) mounting.

The Zeiss Axio Scan Z1 Digital Slide Scanner (Carl Zeiss Microscopy, Germany) was used to acquire whole slide brightfield images. The same scanning profile was used to image the ICH, ICH CAA and control sections using a Plan-Apochromat 20x/0.8 M27 objective.

### Bioimage analysis

Multiplexed immunolabelled tissue sections were imaged in a tiled manner using the ZEISS Axio Scan.Z1 slide scanner. Slides were coded, and researchers were blind to the subject groups (e.g. ICH, ICH CAA, and controls).

An area of 25 mm^2^ from each tissue section was analysed using Indica HALO (v3.6.4134) and HALO AI (v3.6.4134) image analysis software (Indica Labs, San Diego).

Red blood cells (RBCs) were identified based on their biconcave disc shape and autofluorescence in the FITC channel. HALO AI DenseNet v2 and HALO AI Nuclei seg were used to discriminate red blood cells (RBCs) from background and MAP2 positive objects in the FITC channel, respectively. Next, the HALO RandomForest classifier tool was used to identify five classes: background (BG), red blood cells (RBCs; FITC), neurons (microtubule-associated protein 2; MAP2; FITC), astrocytes (glial fibrillary acidic protein; GFAP; Cy5), and microvasculature (endoglin; CD105; brown). Regions of haemorrhage were classified by concentrations of RBCs located outside CD105 positive blood vessels. To train the model, each classifier was created by labelling each feature/cell type associated with the corresponding fluorescence channel or chromogen colour within numerous sample areas. Next, each class was refined and verified using tissue samples across patient groups to ensure that each classification identified the appropriate cell type in each tissue section.

Next, the density of kisspeptin immunoreactivity (Cy3) was measured using the HALO Area Quantification (v2.3.4) module. The baseline threshold for kisspeptin immunoreactivity (Cy3 intensity > 1000) was set using negative control slides. No Cy3 positive objects (e.g. Kiss objects) were identified in the negative controls.

To obtain quantitative data on kisspeptin immunopositivity in relation to neurons (MAP2+), astrocytes (GFAP+), red blood cells (RBC+) and regions of haemorrhage (RBC + minus CD105), the HighPlex FL (v4.2.5) module was utilised by the status of co-localising MAP2, GFAP, RBCs and haemorrhage classifiers.

To understand the spatial relationship of kisspeptin immunoreactivity to regions of haemorrhage, spatial analysis was conducted using HALO’s Infiltration analysis module to identify the numbers of kisspeptin immunoreactive objects within and outside regions of haemorrhage (− 200 mm to + 500 mm).

Next, to assess the spatial distribution of kisspeptin immunoreactivity relative to CD31 + vascular endothelial cells, double immunofluorescence labelling was conducted. Deparaffinisation, antigen retrieval and quenching of endogenous peroxidase was conducted as previously described. After rinsing in PBS, each section was blocked in BB for 45 min. Rabbit anti-kisspeptin antibody (1:15,000; AC564) diluted in BB was applied to each section and slides were incubated at 4 °C overnight. The following morning, slides were washed three times in PBST. Kisspeptin immunoreactivity was detected by incubating sections with donkey anti-rabbit IgG-peroxidase (1:650; Jackson ImmunoResearch Ltd., 711-036-152) at 37 °C for 45 min, followed by three washes in PBST. Kisspeptin immunoreactivity was localised using TSA Plus Cyanine 3 (1:400; Akoya Biosciences US, #NEL744001KT) at 37 °C for 1 h. After incubation, the slides were washed to remove excess TSA Plus Cyanine 3. This was followed by HIER by immersion in 10 mM sodium citrate pH 6.0 using a pressure cooker microwave heating for 17 min. Slides were then cooled and quenched with 3% hydrogen peroxide in methanol for 25 min. After a PBS rinse, mouse monoclonal anti-CD31 antibody (1:75; Agilent US, Clone JC70A) in BB was applied to each section and incubated overnight at 4 °C. Slides were then washed in PBST and CD31 immunoreactivity was detected using the Bond Polymer Refine Detection Kit (Leica, DS9800), which includes an anti-mouse IgG and HRP polymer. Following additional washes in PBST, CD31 was localised with TSA Plus Cyanine 5 (1:400; Akoya Biosciences US, #NEL745001KT) for 10 min. After a wash in PBST, sections were counterstained with Hoechst 33342 (1 µg/ml) for 10 min, rinsed twice in PBST and mounted (Epredia Immu-Mount, Fisher Scientific, 9990412).

The Zeiss Axio Scan Z1 Digital Slide Scanner (Carl Zeiss Microscopy, Germany) was used to acquire whole slide fluorescence images at × 20 objective magnification and data was exported as .czi files. The same scanning profile was used to image the ICH, ICH CAA and sudden death control sections using a Plan-Apochromat 20x/0.8 M27 objective. This scanning profile was created using four different fluorescence channels, H3342 (348/455 nm), FITC (495/519 nm), Cy3 (548/561 nm), Cy5 (650/673).

### Statistical analysis

Statistical analysis was performed using Prism 8 (GraphPad Software, CA, USA) and figures were created with Prism and with HALO’s Figure Maker. Box and whiskers plots display a statistical summary of the mean and 5–95% ranges with each value. All data passed the Shapiro–Wilk test for normality. To compare group means, the Brown-Forsythe and Welch ANOVA test was applied. All statistical tests were two-tailed. Differences were considered statistically significant at *p* < 0.05, while ns refers to not significant.

## Results

### Clinical characteristics of cases and controls

We selected 6 ICH (median age 67.5 (IQR 58.75–78.25); 33% female); 5 ICH CAA (median age 77.0 (IQR 69.50–83.00); 20% female) and 6 sudden death control (median age 71.0 (IQR 58.75–82.50); 33% female) cases. Two of the controls died from acute myocardial infarction, one died from ischemic heart disease, one died from a ruptured aortic aneurysm, and one died from sepsis complications. All ICH cases had evidence of intracerebral haematoma in tissue sections and were histologically confirmed. All cases of ICH CAA were histologically confirmed.

1.1 Kisspeptin is higher in cerebral tissue from ICH patients compared to controls.

**Fig. 1:**
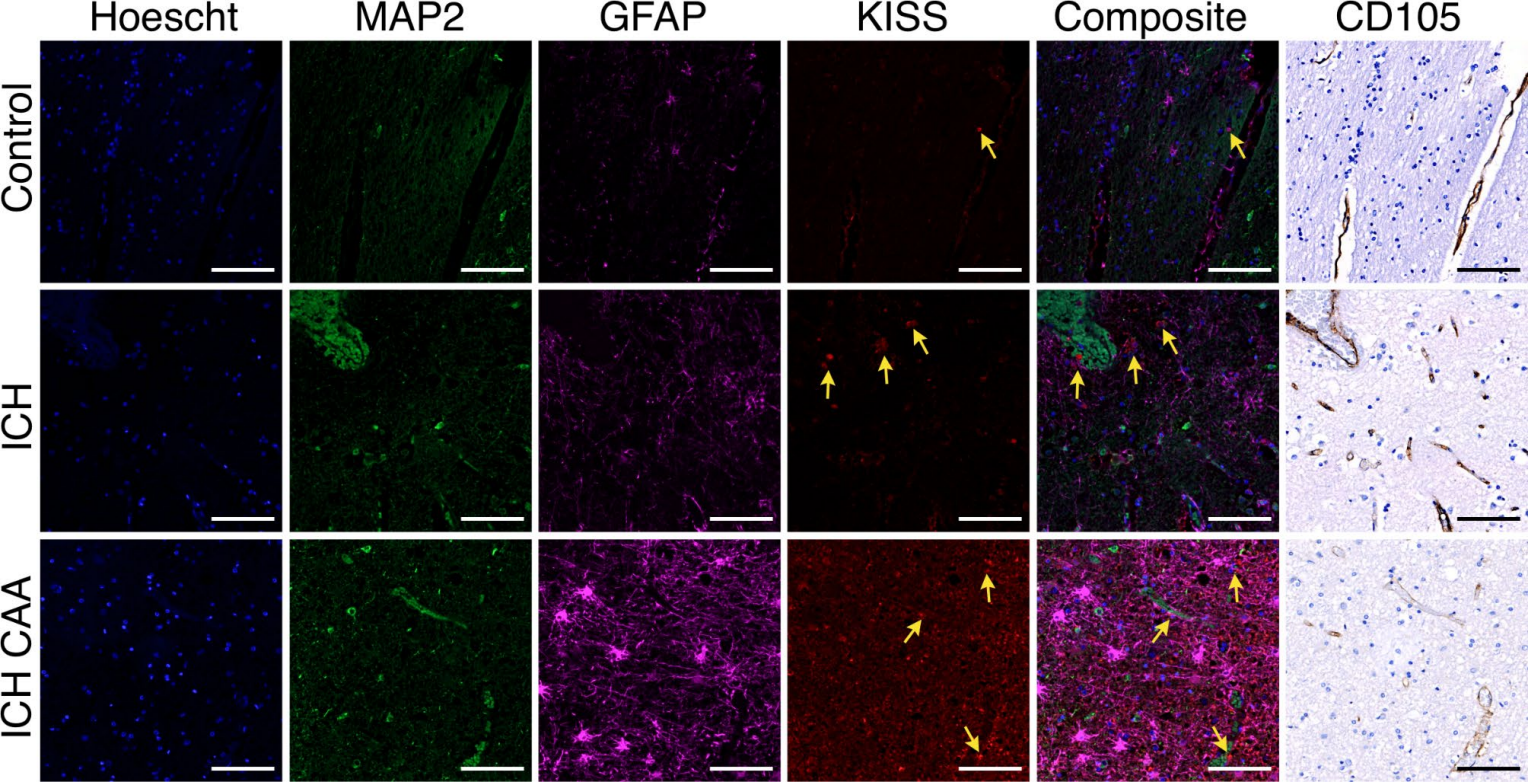
Representative images of kisspeptin immunoreactivity from ICH cases and sudden death controls included in the study. Multiplexed immunofluorescence images from cerebral tissue sections (4 µm thickness) display higher kisspeptin immunoreactivity in intracerebral haemorrhage (ICH) and ICH with cerebral amyloid angiopathy (ICH CAA) cohorts compared to sudden death controls (Control). Hoechst stain (*nuclear marker*) labelled nuclei in the DAPI channel (blue); MAP2 (*neuronal marker*) labelled in the FITC channel (green); GFAP (*astrocyte marker*) labelled in the Cy5 channel (magenta), KISS (*kisspeptin marker*) labelled in the Cy3 channel (red) and composite of all four fluorescence channels. The corresponding immunohistochemistry image for CD105 (*vascular endothelial cell marker*) labelled in brightfield (brown). Scale bars, 100 µm. Yellow arrows highlight KISS-ir cells.

To determine whether kisspeptin was higher in brain tissue from patients who suffered ICH, we measured fluorescence intensity of kisspeptin immunoreactivity using multiplexed immunofluorescence labelling in cerebral tissue sections from both ICH cohorts and compared these to age-matched (sudden-death) controls. KISS-ir was markedly higher in tissue sections from ICH and ICH CAA cohorts compared to controls (Fig. 1).

**Fig. 2:**
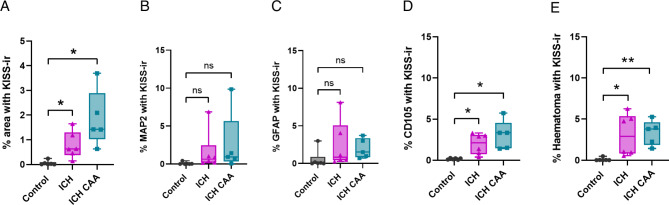
Percentage of tissue area or cell type labelled positive for kisspeptin in ICH, ICH CAA and control cohorts. (**A**) Mean percentage (95% CI) of area positive for kisspeptin (KISS) immunoreactivity (-ir) in cerebral tissue; (**B**) Mean percentage (95% CI) of MAP2 cells positive for KISS-ir; (**C**) Mean percentage (95% CI) of GFAP cells positive for KISS-ir; (**D**) Mean percentage (95% CI) of CD105 cells positive for KISS-ir; (**E**) Mean percentage (95% CI) of haemorrhage positive for KISS-ir. Significance is denoted by asterisks. *p < 0.05; **p < 0.01.

The overall % of each image area that was positive for kisspeptin immunoreactivity was significantly higher in both ICH (mean 0.78%, SD 0.54 KISS-ir per 25 mm^2^; *n* = 6) and ICH CAA cohorts (Fig. 2A, mean 1.85%, SD 1.15 KISS-ir per 25 mm^2^; *n* = 5) compared to controls (mean 0.06%, SD 0.09 KISS-ir per 25 mm^2^; *n* = 6). There was no statistically significant difference between ICH and ICH CAA cohorts.

1.2 There is no difference in kisspeptin labelling in neurons or astrocytes from ICH patients compared to controls.

To establish if the higher kisspeptin immunoreactivity in ICH cohorts was localised to neurons (MAP2+), astrocytes (GFAP+) or cells of the microvasculature (CD105+), we trained HALO Image Analysis Software to classify each cell type based on neuronal, astrocyte and vascular cell markers in each cohort. The fluorescence intensity of kisspeptin immunoreactivity were then quantified (percentage of kisspeptin immunoreactivity) in each cell type. The multiplexed analysis revealed that KISS-ir within neurons was not significantly different between cohorts (Fig. 2B, ICH mean 1.58%, SD 2.62; ICH CAA mean 2.58%, SD 4.10; Control mean 0.09%, SD 0.17; Fig. 2b) nor was KISS-ir in astrocytes (Fig. 2C, ICH mean 2.43%, SD 3.12; ICH CAA mean 1.99%, SD 1.32; Control mean 0.56%, SD 1.20).

1.3 Kisspeptin expression is elevated in the microvasculature and regions of haemorrhage in both ICH and ICH CAA cohorts compared to controls.

The percentage of kisspeptin immunoreactivity in CD105+ microvasculature cells was significantly higher in both ICH and ICH CAA cohorts compared to controls (ICH mean 1.97%, SD 1.25; ICH CAA mean 2.70%, SD 1.82; Control mean 0.18%, SD 0.09; Fig. 2D). A similar trend was observed in CD31+ cells, where kisspeptin immunoreactivity was elevated in ICH cohorts compared to controls (see Supplementary Fig. 2). Notably, no differences were observed in the percentage of kisspeptin immunoreactivity between the two endothelial markers. Furthermore, the percentage of tissue area labelled positive for CD105 or CD31 was comparable across the ICH cohorts and controls (data not shown).

**Fig. 3:**
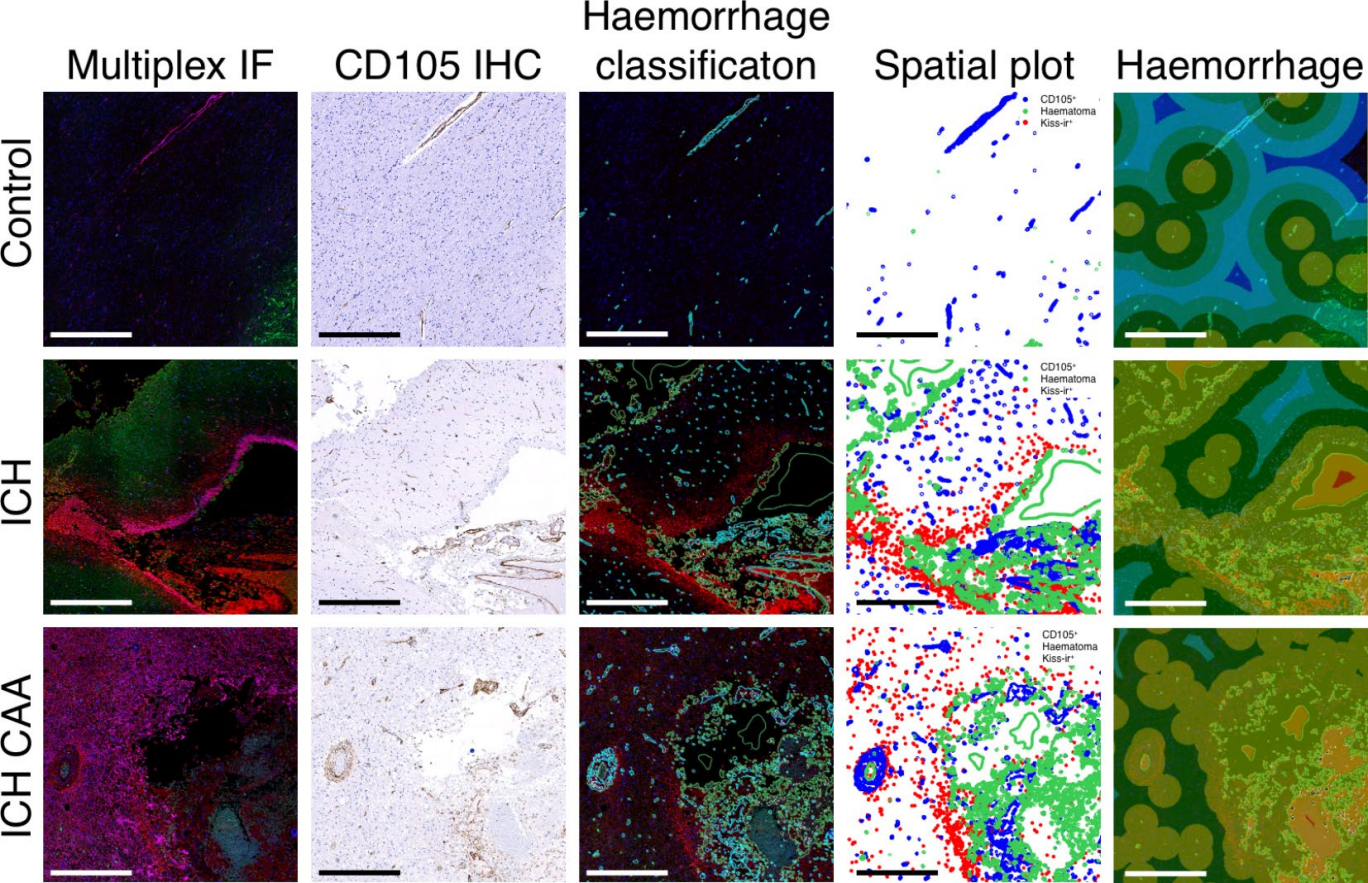
Spatial distribution of kisspeptin immunoreactivity in relation to regions of haemorrhage. Multiplex immunofluorescence images of control, intracerebral haemorrhage (ICH) and ICH with cerebral amyloid angiopathy (ICH CAA) cohorts using KISS (red), GFAP (magenta), MAP2 (green); The corresponding immunohistochemistry image for CD105 marker (brown); The corresponding regions classified as haemorrhage (green) and the microvasculature (cyan); Spatial analysis for KISS-ir objects (red circles), microvasculature cells (CD105+ cells; blue circles) and haematoma red blood cells (green circles); Proximity lines (100 µm bands) are shown for haematoma regions within 200 µm to 500 µm outside. Scale bars, 500 µm.

**Fig. 4:**
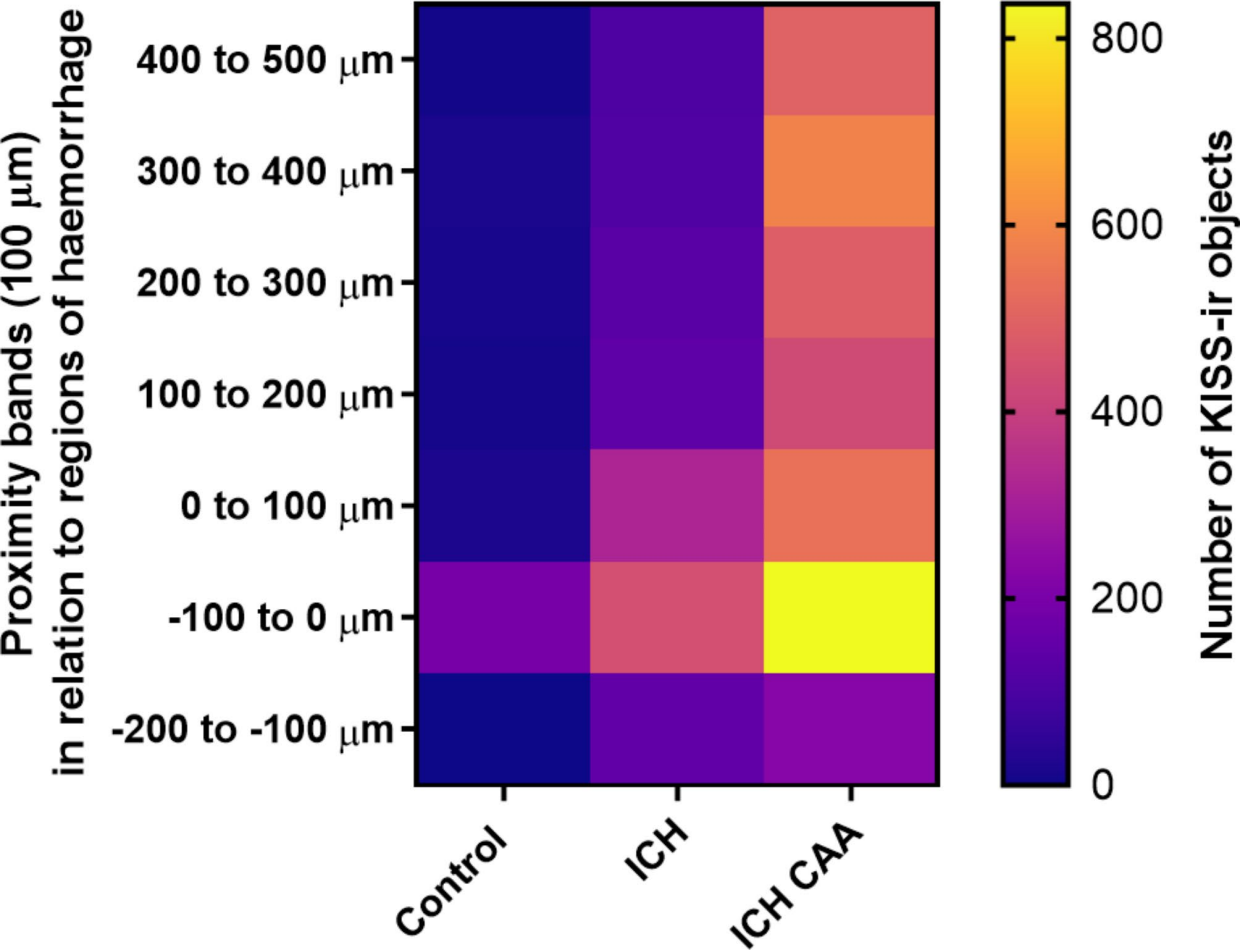
The Spatial Infiltration Heatmap of the distribution of kisspeptin immunoreactive objects in relation to regions of haemorrhage. Proximity bands (100 µm) are shown within 200-µm to 500-µm outside of regions of haemorrhage. The quantity of KISS-ir objects range from 0 to 836 (scale: dark purple to yellow).

To further understand if kisspeptin was localised to the haemorrhage, we used HALO image analysis to classify regions of haemorrhage by identifying areas concentrated with red blood cells positioned outside of the vasculature. In haemorrhagic regions, the expression of kisspeptin was significantly higher in both ICH and ICH CAA compared to control regions in the sudden-death cohort (ICH mean 3.09%, SD 2.53; ICH CAA mean 3.34%, SD 1.51; Control mean 0.11%, SD 0.19; Fig. 2E). Next to understand if kisspeptin immunoreactivity was concentrated within and around regions of haemorrhage, we conducted Spatial Image Analysis. Examples of the tissue classification is shown (See Fig. 3). Displayed on the heatmap (Fig. 4), demonstrates that the largest number of KISS-ir objects are located within 100 µm of haemorrhagic regions in both the ICH and ICH CAA cohorts.

**Fig. 5:**
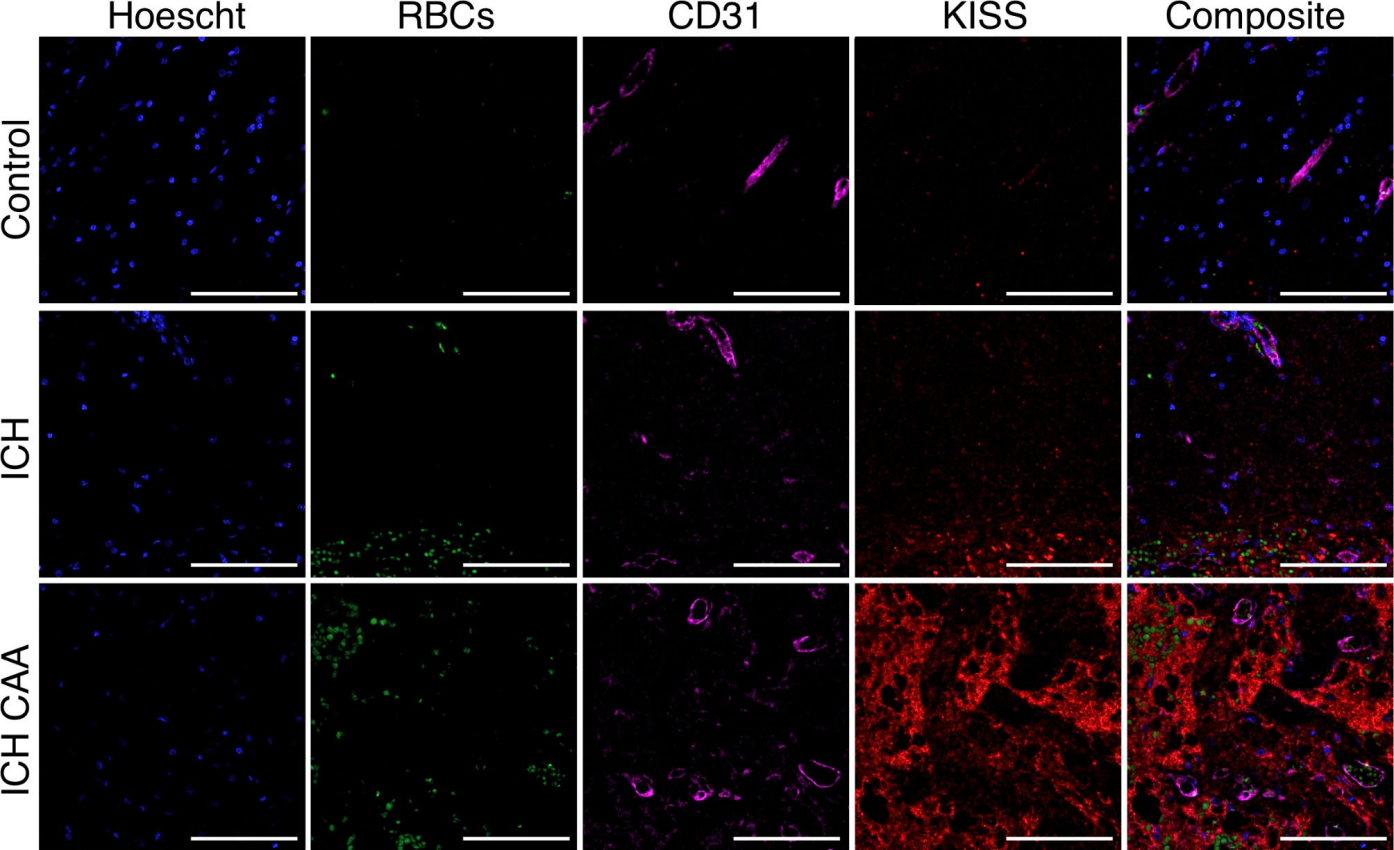
The distribution of kisspeptin immunoreactivity in relation to CD31+ vasculature. Multiplexed immunofluorescence images from cerebral tissue sections show elevated kisspeptin immunoreactivity near red blood cells in proximity to CD31+ vasculature in both intracerebral haemorrhage (ICH) and ICH with cerebral amyloid angiopathy (ICH CAA) cohorts, compared to sudden death controls (Control). Hoechst stain (*nuclear marker*) labelled nuclei in the DAPI channel (blue); red blood cell autofluorescence in the FITC channel (green); CD31 (*vasculature endothelial marker*) labelled in the Cy5 channel (magenta), and KISS (*kisspeptin marker*) labelled in the Cy3 channel (red); and a composite of all four fluorescence channels. Scale bars, 100 µm.

To investigate the localisation of kisspeptin within CD31+ vasculature and its relationship to red blood cells, we performed multiplexed immunofluorescence labelling for kisspeptin and PECAM-1 (CD31) (Fig. 5). The results showed that kisspeptin immunoreactivity was not confined to CD31+ vascular endothelial cells but was primarily localised to regions with a high density of red blood cells (i.e., haemorrhagic regions).

## Discussion

To our knowledge this is the first case-control study of kisspeptin after ICH stroke. To better understand whether kisspeptin is involved in stroke pathophysiology, we tested the hypothesis that kisspeptin immunoreactivity is higher in the brain from patients that suffered either ICH or ICH associated with CAA, compared to controls (who died suddenly from other causes). The results showed: (1) the percentage of area immunoreactive for kisspeptin was higher in both ICH cohorts compared to controls; (2) There was no difference in kisspeptin immunoreactivity associated with neurons or astrocytes after ICH in both cohorts compared to controls; (3) Kisspeptin immunoreactivity was higher in cells of the microvasculature (CD105+) in both ICH cohorts compared to controls; (4) Kisspeptin immunoreactivity was highest within haemorrhagic regions in both ICH cohorts compared to control regions in patients who died suddenly from other means. Taken together, our results suggest that kisspeptin is significantly higher in post-mortem tissue after ICH. The concentration of kisspeptin immunoreactivity to the haemorrhagic regions and the microvasculature suggests that kisspeptin is expressed from the local vasculature or is recruited to regions of haemorrhage.

In humans, earlier work found that kisspeptin circulates in blood plasma and KISS and KISS1R immunoreactivity was localised to the coronary artery and umbilical vein^18^. This suggests an endothelial source, potentially regulating vascular tone through a local paracrine mechanism. Furthermore, KISS-54, KISS-13 and KISS-10 all have equipotent vasoconstrictive potential on coronary artery and umbilical vein vessel rings ex vivo, and have a comparable potency to angiotensin II in myograph studies^18^. Another study demonstrated vasoconstrictive actions for KISS-10 in the peripheral vasculature and proposed that kisspeptins can influence microvascular events such as increasing plasma extravasation and reducing peripheral blood flow^25^.

ICH is associated with arteriole rupture and profuse parenchymal haemorrhage resulting in subsequent haematoma formation. The mass effect of primary bleeding and haematoma expansion increases intracranial pressure and results in neuronal cell death^26,27^. Haematoma growth begins immediately after haemorrhage and is an independent determinant of mortality and poor neurological outcome following ICH^28^. In order to minimise cell death, it is crucial to restrict further haematoma formation post-stroke. The increase in kisspeptins following ICH may stimulate vasoconstriction and reduce blood flow to directly stem the haemorrhage and mitigate hematoma development. A recent preclinical rat study demonstrated that kisspeptin and its receptor are rapidly upregulated after SAH, with levels increasing at 3 h and peaking at 24 h^19^. Knockdown of *Kiss1* was shown to exacerbate neurobehavioural deficits after SAH compared to controls whereas exogenous treatment with Kiss-54, an hour after SAH, led to attenuated brain oxidative stress, decreased neuronal degeneration and reduced neurological deficits through activating the Kiss1r/ARRB2/AKT/GSK3β signalling pathway^19^. Interestingly, administration of Kiss within an hour post-stroke has been shown to improve spatial learning and memory and alleviate memory impairment induced by beta-amyloid in rodents^29,30^ and an earlier study suggested that kisspeptin was neuroprotective against neuronal damage caused by oxidative stress^31^. In addition, a recent mouse study found that administration of Kiss-10 prior to the induction of cerebral ischemia led to an increase in glial cells and a reduction in brain dopamine levels suggesting that kisspeptin may play a role in regulating brain neurotransmitter release and modulating inflammation at the site of injury^32^. Interestingly in our study, high kisspeptin immunoreactivity was correlated with an increase in GFAP (astrocyte) labelling around regions of haemorrhage (data not shown). However, high kisspeptin immunoreactivity was observed days after ICH indicating a more prolonged expression. Further research is required to establish how kisspeptin within the injury microenvironment changes with the progression of stroke, particularly in relation to secondary brain injury and the longer-term neuroinflammatory response. Such studies could lay the ground work for exploring kisspeptin as a novel therapeutic target for stroke.

In our study, kisspeptin is concentrated to sites of haemorrhage and would likely enter the general circulation after ICH due to breakdown in the blood-brain barrier (BBB). However, neuropeptides tend to have short half-lives in the circulation, with kisspeptin-10 reported to have a half-life of 3.8 min^33^ and kisspeptin-54 a half-life of 27.6 min^34^. Because kisspeptins are cleared rapidly from the circulation, it is tempting to think that elevated kisspeptin after ICH might reflect a dynamic process underlying primary brain injury, potentially holding prognostic value. Furthermore, it is important to determine whether the magnitude of kisspeptin detection is dependent on haemorrhage severity, or whether this correlation is complicated by a potential neuroprotective role. As our study investigated kisspeptin immunoreactivity in relation to ICH stroke specifically, further work is required to establish if and how KISS may be elevated following ischaemic stroke. Differential expression of KISS following ischaemic and haemorrhagic stroke may provide insights into the mechanisms underpinning KISS upregulation and warrants its further study as a diagnostic tool. Regardless, kisspeptin would be clinically useful as a biomarker if its presence is sensitive enough to differentiate ICH from other stroke mimics, possibly reducing patient exposure to ionising radiation. Whilst this study clearly demonstrates an association between increased kisspeptin immunoreactivity and ICH, to fully appreciate the utility of kisspeptin as a prospective biomarker it will be important to establish to what extent kisspeptin is expressed directly by the intraparenchymal endothelium, and whether kisspeptin may be able to enter from or leave to the peripheral vasculature through an injured BBB. The relationship between kisspeptin in the peripheral vasculature and ICH may provide clinicians vital information and will be the subject of future studies.

This study had several strengths. We minimised selection bias by randomly selecting ICH, ICH CAA and control samples with age and sex distributions similar to those of patients incident with ICH in our population. To maximise power, we used an approximate 2:1 ratio of ICH cases to controls. We identified ICH-free controls who died suddenly from the same population, ensuring their brain tissue was acquired and processed using the same standardised protocol. During image analysis, investigators were blinded to groups assignments, and quantifications were automated using the HALO Image Analysis Platform, minimising potential bias.

While we found a significant difference in kisspeptin immunoreactivity between stroke patients and controls, the overall study sample size was small. Future studies with larger sample sizes may be able to discriminate differences in KISS expression between ICH and ICH CAA cohorts.

As shown in Table 1, samples were taken from a range of patients and controls. We attempted to minimise confounding factors by matching our cases for age, sex and ICH location. Although baseline conditions for sex and age were similar between cases and controls, the ICH CAA cohort had mainly lobar ICH locations, whereas the ICH cohort had more samples with deep ICH locations. This likely reflects the aetiology of CAA associated with ICH, which typically results in haemorrhages in lobar and superficial brain areas, in contrast to ICH associated with hypertension, which commonly results in haemorrhages in deep grey matter such as the basal ganglia, thalamus and brain stem^35^. Additionally, the time intervals from ICH onset to death were quite broad in the ICH CAA cohort (3–1216 days). However, our exploratory analysis did not find any association between kisspeptin immunoreactivity and either ICH location or the time to death post-ICH. Whilst we found that kisspeptin immunoreactivity was increased in both stroke cohorts, ICH CAA showed a non-significant trend of increased kisspeptin immunoreactivity in microvasculature cells as well as the haemorrhage site when compared to the ICH group. Further work with a greater sample size is required to determine if this association is statistically significant.

In summary, our results indicate that kisspeptin immunoreactivity is significantly higher in ICH brain tissue with and without CAA. Kisspeptin localised to cells of the microvasculature would likely enter the circulation after breakdown of the BBB after stroke. Altogether these results emphasise the need for further basic and translational research to understand the molecular mechanisms underlying kisspeptin elevation in cerebral tissue from patients who suffered ICH stroke. This might serve as the foundation for further exploration of kisspeptin as a potential therapeutic target and/or biomarker for ICH stroke.

## Electronic Supplementary Material

Below is the link to the electronic supplementary material.

Supplementary Material 1

## Data Availability

Data is provided within the manuscript or supplementary information files.

## References

[1] Ikram, M. A., Wieberdink, R. G. & Koudstaal, P. J. International epidemiology of intracerebral hemorrhage. *Curr. Atheroscler. Rep.* **14**, 300–306 (2012). 22538431 10.1007/s11883-012-0252-1 PMC3388250

[2] Perna, R. & Temple, J. Rehabilitation outcomes: Ischemic versus hemorrhagic strokes. *Behav. Neurol.* **2015**, 891651 (2015). 26246694 10.1155/2015/891651 PMC4515256

[3] Rincon, F., Lyden, P. & Mayer, S. A. Relationship between temperature, hematoma growth, and functional outcome after intracerebral hemorrhage. *Neurocrit. Care* **18**, 45–53 (2013). 23001769 10.1007/s12028-012-9779-9

[4] van Asch, C. J. et al. Incidence, case fatality, and functional outcome of intracerebral haemorrhage over time, according to age, sex, and ethnic origin: A systematic review and meta-analysis. *Lancet Neurol.* **9**, 167–176 (2010). 20056489 10.1016/S1474-4422(09)70340-0

[5] Tang, D., Chen, X., Kang, R. & Kroemer, G. Ferroptosis: Molecular mechanisms and health implications. *Cell Res.* **31**, 107–125 (2021). 33268902 10.1038/s41422-020-00441-1 PMC8026611

[6] Sacco, S. et al. Declining incidence of intracerebral hemorrhage over two decades in a population-based study. *Eur. J. Neurol.* **23**, 1627–1634 (2016). 27456069 10.1111/ene.13099

[7] Béjot, Y. et al. Temporal trends in early case-fatality rates in patients with intracerebral hemorrhage. *Neurology* **88**, 985–990 (2017). 28159886 10.1212/WNL.0000000000003681

[8] Biffi, A. & Greenberg, S. M. Cerebral amyloid angiopathy: A systematic review. *J. Clin. Neurol. Seoul Korea* **7**, 1–9 (2011). 10.3988/jcn.2011.7.1.1 PMC3079153 21519520

[9] Viswanathan, A. & Greenberg, S. M. Cerebral amyloid angiopathy in the elderly. *Ann. Neurol.* **70**, 871–880 (2011). 22190361 10.1002/ana.22516 PMC4004372

[10] Reijmer, Y. D., van Veluw, S. J. & Greenberg, S. M. Ischemic brain injury in cerebral amyloid angiopathy. *J. Cereb. Blood Flow Metab.* **36**, 40–54 (2016). 25944592 10.1038/jcbfm.2015.88 PMC4758563

[11] Kozberg, M. G., Perosa, V., Gurol, M. E. & van Veluw, S. J. A practical approach to the management of cerebral amyloid angiopathy. *Int. J. Stroke* **16**, 356–369 (2021). 33252026 10.1177/1747493020974464 PMC9097498

[12] Anathhanam, S. & Hassan, A. Mimics and chameleons in stroke. *Clin. Med.* **17**, 156–160 (2017). 10.7861/clinmedicine.17-2-156 PMC6297626 28365629

[13] Birenbaum, D., Bancroft, L. W. & Felsberg, G. J. Imaging in Acute Stroke. *West. J. Emerg. Med.* **12**, 67–76 (2011). 21694755 PMC3088377

[14] de Roux, N. et al. Hypogonadotropic hypogonadism due to loss of function of the KiSS1-derived peptide receptor GPR54. *Proc. Natl. Acad. Sci.* **100**, 10972–10976 (2003). 12944565 10.1073/pnas.1834399100 PMC196911

[15] Funes, S. et al. The KiSS-1 receptor GPR54 is essential for the development of the murine reproductive system. *Biochem. Biophys. Res. Commun.* **312**, 1357–1363 (2003). 14652023 10.1016/j.bbrc.2003.11.066

[16] Seminara, S. B. et al. The *GPR54* Gene as a Regulator of Puberty. *N. Engl. J. Med.* **349**, 1614–1627 (2003). 14573733 10.1056/NEJMoa035322

[17] Topaloglu, A. K. et al. Inactivating *KISS1* Mutation and Hypogonadotropic Hypogonadism. *N. Engl. J. Med.* **366**, 629–635 (2012). 22335740 10.1056/NEJMoa1111184

[18] Mead, E. J., Maguire, J. J., Kuc, R. E. & Davenport, A. P. Kisspeptins are novel potent vasoconstrictors in humans, with a discrete localization of their receptor, G protein-coupled receptor 54, to atherosclerosis-prone vessels. *Endocrinology* **148**, 140–147 (2007). 17023533 10.1210/en.2006-0818

[19] Huang, Y. et al. Kisspeptin-54 attenuates oxidative stress and neuronal apoptosis in early brain injury after subarachnoid hemorrhage in rats via GPR54/ARRB2/AKT/GSK3β signaling pathway. *Free Radic. Biol. Med.* **171**, 99–111 (2021). 33989759 10.1016/j.freeradbiomed.2021.05.012 PMC8388553

[20] Millar, T. et al. Tissue and organ donation for research in forensic pathology: The MRC sudden death brain and tissue bank. *J. Pathol.* **213**, 369–375 (2007). 17990279 10.1002/path.2247

[21] Franceschini, I. et al. Kisspeptin immunoreactive cells of the ovine preoptic area and arcuate nucleus co-express estrogen receptor alpha. *Neurosci. Lett.* **401**, 225–230 (2006). 16621281 10.1016/j.neulet.2006.03.039

[22] Matsubara, S., Bourdeau, A., terBrugge, K. G., Wallace, C. & Letarte, M. Analysis of endoglin expression in normal brain tissue and in cerebral arteriovenous malformations. *Stroke* **31**, 2653–2660 (2000). 11062290 10.1161/01.str.31.11.2653

[23] Nassiri, F. et al. Endoglin (CD105): A review of its role in angiogenesis and tumor diagnosis, progression and therapy. *ANTICANCER Res.* (2011). 21737653

[24] Privratsky, J. R. & Newman, P. J. PECAM-1: Regulator of endothelial junctional integrity. *Cell Tissue Res.* **355**, 607–619 (2014). 24435645 10.1007/s00441-013-1779-3 PMC3975704

[25] Sawyer, I. et al. The vasoactive potential of Kisspeptin-10 in the peripheral vasculature. *PLoS ONE* **6**, e14671 (2011). 21347414 10.1371/journal.pone.0014671 PMC3036649

[26] Aronowski, J. & Zhao, X. Molecular pathophysiology of cerebral hemorrhage: Secondary brain injury. *Stroke J. Cereb. Circ.* **42**, 1781–1786 (2011). 10.1161/STROKEAHA.110.596718 PMC3123894 21527759

[27] Prabhakaran, S. & Naidech, A. M. Ischemic brain injury after intracerebral hemorrhage. *Stroke* **43**, 2258–2263 (2012). 22821611 10.1161/STROKEAHA.112.655910

[28] Davis, S. M. et al. Hematoma growth is a determinant of mortality and poor outcome after intracerebral hemorrhage. *Neurology* **66**, 1175–1181 (2006). 16636233 10.1212/01.wnl.0000208408.98482.99

[29] Ebrahimi Khonacha, S., Janahmadi, M. & Motamedi, F. Kisspeptin-13 improves spatial memory consolidation and retrieval against amyloid-β pathology. *Iran. J. Pharm. Res. IJPR* **18**, 169–181 (2019). 32802097 10.22037/ijpr.2019.112199.13599 PMC7393055

[30] Jiang, J. H. et al. Kisspeptin-13 enhances memory and mitigates memory impairment induced by Aβ1–42 in mice novel object and object location recognition tasks. *Neurobiol. Learn. Mem.* **123**, 187–195 (2015). 26103138 10.1016/j.nlm.2015.05.010

[31] Akkaya, H., Kilic, E., Eyuboglu Dinc, S. & Yilmaz, B. Postacute effects of Kisspeptin-10 on neuronal injury induced by L-methionine in rats. *J. Biochem. Mol. Toxicol.* **28**, 373–377 (2014). 24863683 10.1002/jbt.21573

[32] Akkaya, H., Sumer, E., Kutlu, S., Solak, H. & Yilmaz, B. What is the protective effect of preischemic kisspeptin-10 administration against ischemia/reperfusion injury of striatum on mice?. *Turk. J. Med. Sci.* **52**, 1532–1542 (2022). 36422497 10.55730/1300-0144.5493 PMC10395688

[33] Jayasena, C. N. et al. The effects of Kisspeptin-10 on reproductive hormone release show sexual dimorphism in humans. *J. Clin. Endocrinol. Metab.* **96**, E1963–E1972 (2011). 21976724 10.1210/jc.2011-1408 PMC3232613

[34] Dhillo, W. S. et al. Kisspeptin-54 stimulates the hypothalamic-pituitary gonadal axis in human males. *J. Clin. Endocrinol. Metab.* **90**, 6609–6615 (2005). 16174713 10.1210/jc.2005-1468

[35] Chen, S.-J. et al. Advances in cerebral amyloid angiopathy imaging. *Ther. Adv. Neurol. Disord.* **12**, 1756286419844113 (2019). 31105769 10.1177/1756286419844113 PMC6501479

