# Supplementary figures and images for: Kisspeptin is elevated in the brain after intracerebral haemorrhagic stroke
Source: Sci Rep. 2024 Dec 30;14:32046. doi: 10.1038/s41598-024-83514-0 (PMC11685885; doi:10.1038/s41598-024-83514-0)

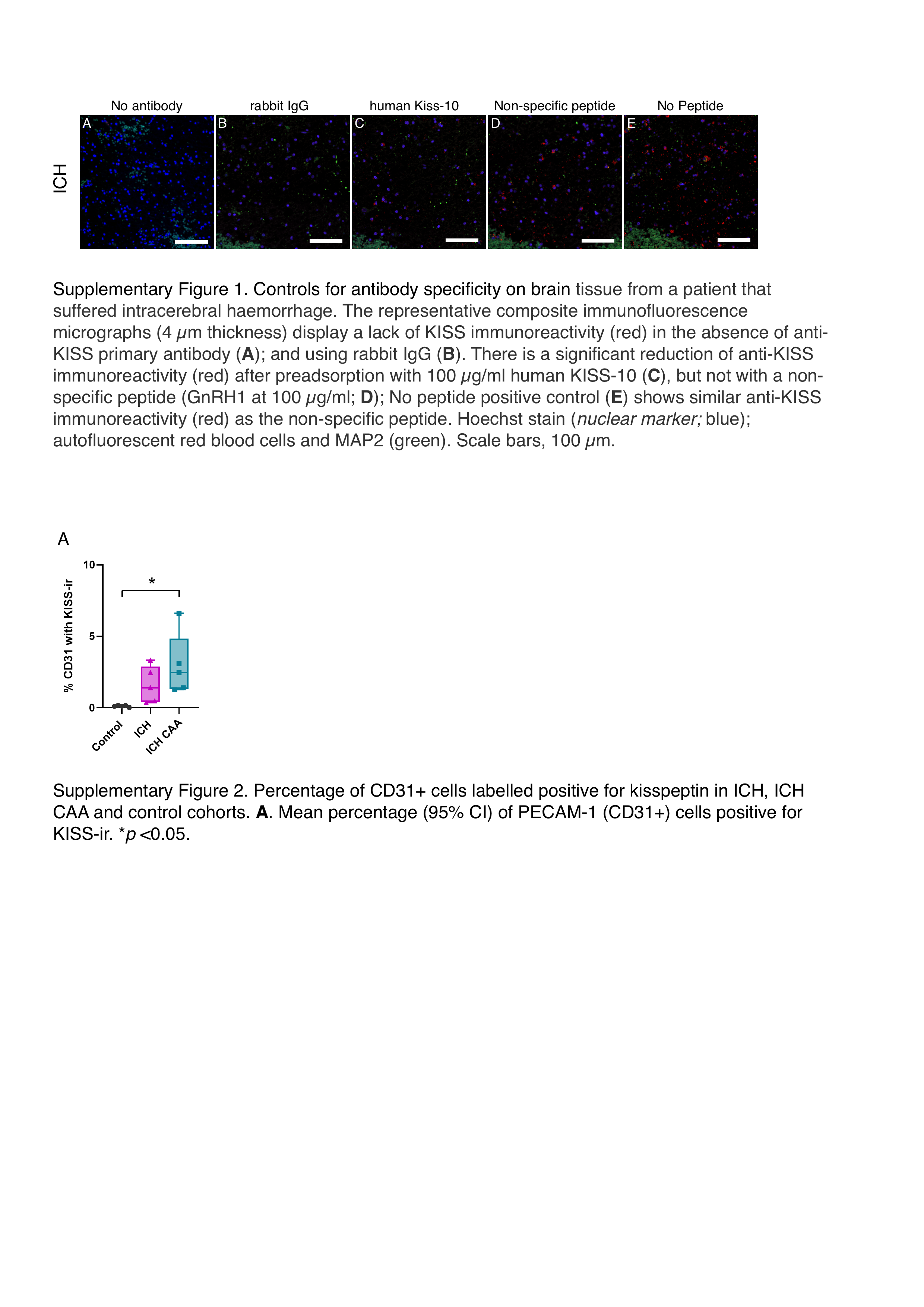

Supplement: Supplementary file 1 — Supplementary Material 1 [file 41598_2024_83514_MOESM1_ESM.tiff]
